# Evaluation of Changes in Hemorheological Variables in Multiple Sclerosis Patients

**DOI:** 10.31661/gmj.v13i.3513

**Published:** 2024-12-31

**Authors:** Mahdi Yaseliani, Abdolreaza Naser Moghadasi, Mohammad Ali Sahraian, Mehrdad Karimi, Mahdi Alizadeh Vaghasloo, Hossein Rezaeizadeh

**Affiliations:** ^1^ Department of Traditional Medicine, School of Persian Medicine, Tehran University of Medical Sciences, Tehran, Iran; ^2^ Multiple Sclerosis Research Center, Neuroscience Institute, Tehran University of Medical Sciences (TUMS), Tehran, Iran; ^3^ Multiple Sclerosis Research Center, Neuroscience Institute, Tehran University of Medical Sciences, Neurology Department, Sina Hospital, Tehran, Iran

**Keywords:** Multiple Sclerosis, Hemorheology, Cerebral Blood Flow, Red Blood Cell

## Abstract

Background: Multiple sclerosis (MS) is a neurodegenerative condition primarily attributed to immune system dysregulation. However, emerging evidence suggests that additional factors, such as neurodegeneration independent of immune processes, may also contribute to MS pathology. Given the significant cerebral hypoperfusion observed in MS patients from the early to advanced stages of the disease, investigating hemorheology or blood rheology, which involves studying blood flow properties and plasma protein compounds, can contribute to understanding the underlying pathology of MS. This study aims to evaluate changes in hemorheological variables in MS patients, which may offer a better understanding of the disease’s progression and its impact on blood flow dynamics.Materials and Methods: In this study, we assessed the modifications in key factors impacting hemorheology in articles related to MS. Some keywords including MS, Blood Viscosity, Hemorheology, and brain perfusion were searched in PubMed, Google Scholar, and Scopus. The searches were limited to studies published in English languages from 2000 to 2023. Results: Among the 110 articles found in the search, finally, 35 articles were included in the review. In some studies, patients with MS were examined for rheological blood properties and demonstrated an appreciable increase in blood viscosity. Furthermore, declines in cerebral blood volume and cerebral blood flow in MS are linked to the deterioration of physical disability. In our investigation, we focused on the key factors influencing hemorheology and examined their variations in the articles about patients with MS. Conclusion: The reduction of tissue blood perfusion caused by changes in blood hemorheology can be considered as one of the causes of the development or exacerbation of MS, but to estimate hemorheological changes in MS, we need to conduct more detailed studies on humans, which we hope will provide new solutions for the therapists of this disease.

## Introduction

Multiple sclerosis (MS) is a disease impacting the central nervous system (CNS) in various ways. It is characterized by demyelination and neurodegeneration, presenting with various clinical manifestations [8]. The pathogenesis of MS is intricate, involving T- and B-cell mechanisms, and its diverse manifestations make the underlying cause unclear [1]. While the widely accepted hypothesis attributes MS to immune system dysregulation, resulting in immune cell infiltration into the CNS and subsequent demyelination, axonal damage, and neurodegeneration [9], some observations suggest that MS pathology may not solely arise from primary immune dysregulation [2][3].

There are indications that plaque formation may have origins beyond destructive cell-mediated immunity targeting myelin or oligodendrocyte antigens.

Hemorheology, the study of blood flow properties, is directly influenced by factors such as hematocrit levels, plasma viscosity, and erythrocyte deformability, all of which regulate the ease with which blood flows through small vessels. In MS, reduced blood flow could impair oxygen and nutrient delivery to CNS tissues, thereby worsening neuroinflammation, oxidative stress, and neuronal injury. This disruption in microcirculation may also promote the formation of hypoxic zones, which are known to exacerbate axonal degeneration and contribute to lesion formation.

Altered blood rheology mechanisms can exacerbate MS symptoms by impairing the brain’s capacity to maintain adequate perfusion, thereby increasing the risk of further neurological damage. Understanding these mechanisms could be pivotal in explaining the heterogeneous clinical manifestations of MS and may point toward novel therapeutic strategies aimed at improving cerebral perfusion.

Perfusion-weighted imaging studies have revealed widespread cerebral hypoperfusion in MS patients, persisting from the early stages to more advanced disease phases [4]. The flow rates through blood vessels are influenced by various physical factors, including the geometric characteristics of the vessels, the pressure generated by the heart driving the flow, and the rheological properties of the blood [10].

Hemorrology or blood rheology is the study of blood flow properties and its plasma protein compositions. Normal tissue perfusion occurs within certain rheological limits and any change can significantly lead to the disease state [11].

The rheological characteristics of blood, a fluid with two phases, are influenced by various factors, such as the hematocrit value indicating the relative volume of each phase, plasma composition, and the properties of cellular elements. Consequently, alterations in blood rheology mechanisms are closely connected to these factors [5]. In this study, we assessed the modifications in key factors affecting hemorheology, as discussed in articles related to MS.

In this article, we evaluated the changes of the most important factors affecting hemorheology in the articles related to MS.

## Materials and Methods

In this literature review, keywords including MS, blood viscosity, hemorheology, and brain perfusion were searched in PubMed, Google Scholar, and Scopus databases. The searches were restricted to studies published in English between 2000 and 2023. We systematically screened titles and abstracts for relevance. Full-text articles were then reviewed, and studies were included if they provided quantitative data on hemorheological parameters, including hematocrit levels, plasma viscosity, erythrocyte deformability, and cerebral blood flow in MS patients. Studies were excluded if full texts were unavailable or if they did not provide primary data related to hemorheology. Data extracted from the included studies involved variables such as blood viscosity, hematocrit values, plasma composition, cerebral perfusion rates, and alterations in cellular elements that contribute to blood rheology. These extracted data were categorized and coded in Microsoft Excel for analysis. The references were managed using EndNote software.

Search Strategy

• The initial search phase involved using the combination of the selected keywords with Boolean operators ("AND" and "OR") to capture a broad set of relevant articles. For example, search strings such as "Multiple Sclerosis AND Hemorheology" and "Blood Viscosity AND Brain Perfusion AND Multiple Sclerosis" were applied across the databases.

• To ensure the inclusion of the most recent and relevant studies, we refined the search by applying filters to limit the results to articles published in English between the years 2000 and 2023. We also restricted the scope to research articles, reviews, and clinical trials.

Inclusion and Exclusion Criteria

**Figure-1:**
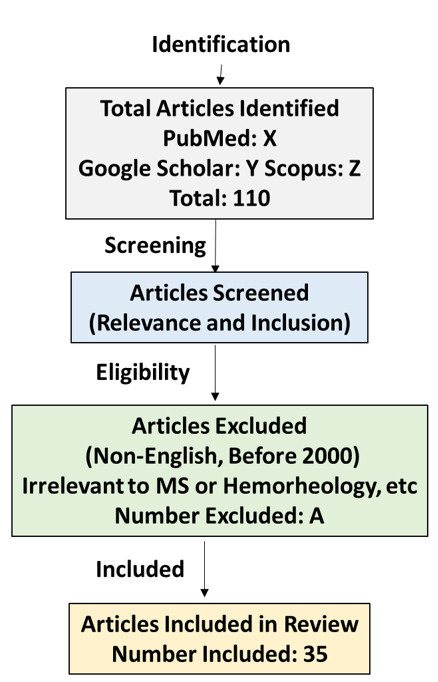
Figure 1. Overview of the flow from the initial identification of articles through screening, eligibility, and final inclusion in the review.

• Inclusion criteria: Only studies investigating hemorheological factors such as blood viscosity, hematocrit, or cerebral perfusion in MS patients were included. Research articles, reviews, and clinical trials related to the interaction between blood flow properties and MS pathology were considered.

• Exclusion criteria: Studies not directly focused on MS, studies not involving hemorheological measurements, and articles published in languages other than English were excluded. Animal studies were also excluded unless they directly contributed to understanding human MS pathology. (Figure-1)

Classification of Blood Constituents affecting Hemorheology

1. Cellular phase (Formed Elements)

• Erythrocytes (RBC)

• Leukocytes (WBC)

• Platelets (PLT)

2. liquid phase (Plasma)

• Proteins

• Ions

• Metabolic Molecules

## Results

**Figure-2:**
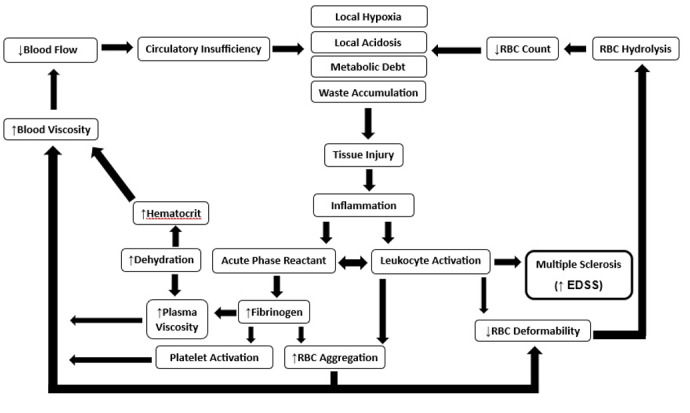
Figure 2. The relation of blood factors affecting hemorrology in the articles of MS patients

The primary role of blood is to supply essential nutrients to all body tissues and eliminate waste [12]. It is well-established that the rates of blood circulation within blood vessels are influenced by various physical factors. These factors encompass the geometric attributes of the vessels, the pressure exerted by the heart to propel the flow, and, notably, the rheological properties of the blood, which constitute the focus of our discussion [10].

Studies have shown that impaired tissue blood flow plays a crucial role in the onset of neurodegenerative conditions. Widespread areas of hypoperfusion are known in Alzheimer and Parkinson disease compared with controls [13].

Decreases in cerebral blood volume and cerebral blood flow in MS have been linked to deterioration in physical disability [7]. This association has been consistently noted in studies examining disability and functional test scores [14][15]. Numerous investigations consistently highlight the relationship between cognitive decline, diminished perfusion parameters [16][17], and fatigue in MS [16][18]. In the context of MS, vascular congestion, accompanied by thrombi in small veins and focal ischemic damage, is believed to play a pivotal role in the development of CNS plaques [19]. Early-stage MS observations indicate that demyelination plaques are concentrated around small veins characterized by lower shear rates and higher blood viscosity [20].

In a study involving 45 MS patients, an examination of rheological blood properties revealed notable differences compared to controls. The examined group exhibited increased plasma viscosity, accelerated red blood cell aggregation, and a significant 26.2% of patients displayed elevated blood viscosity. These changes are believed to contribute to the deterioration of microcirculation and exacerbate the demyelination process [6]. Roizin et al. observed blood sludging within conjunctival vessels of MS patients, proposing that this sludge may obstruct capillaries supplying nervous tissue. These findings of congestion, stasis, and sludging provide evidence of impaired microvascular blood flow in MS, consistent with expectations if blood rheology was abnormal [21]. Since there is no study that directly examines the role of hemorheological variables in patients with MS, we have selected the most important factors affecting hemorrology and observed their changes in the articles of MS patients as follow.

1.The effects of changes in blood components on hemorheology

1.1. Cellular phase

1.1.1. RBC

The normal hematocrit range varies between genders, with men typically ranging from 40 to 50%, and women from 36 to 46%. In contrast, the combined percentage of leukocytes and platelets is relatively low, approximately 1%. The elevated concentration of red blood cells is the primary factor that renders them highly significant from a hemorheological perspective [12].

Another reason for the importance of red blood cells in terms of rheology is due to its different physical properties. Firstly, they have unusual shape as biconcave discs and therefore have the capacity to align with the flow direction. Secondly, red blood cells exhibit considerable flexibility and deformability in response to shear forces. Thirdly, they tend to loosely adhere to each other, forming rouleaux, particularly influenced by plasma proteins, notably fibrinogen. Fourthly, their composition includes hemoglobin, influencing the rate at which they deform under shear forces. These characteristics collectively contribute to imparting blood with significantly higher viscosity compared to plasma alone and bestow upon it notable shear-thinning properties [12].

If the number of red blood cells becomes more than normal, the viscosity increases as a result and ultimately leads to a decrease in tissue perfusion.

The approximate measurement of the relation between blood viscosity and hematocrit is typically determined by the following formula:

log (viscosity)=k1 + k2 (hematocrit) where k1 and k2 are shear rate dependent. For healthy blood, the viscosity at shear rates of 0.277 s-¹ and 128.5 s-¹ typically falls within the ranges of 39±4 and 4.3±0.2 mPa.s, respectively, for females, and 48±6 and 4.7±0.2 mPa.s, respectively, for males [22]. The variation between genders is attributed to the normal lower hematocrit levels observed in women.

Hematocrit serves as the most factor influencing blood viscosity in situations of bulk flow, such as in large diameter vessels or viscometers with substantial geometries. Nonetheless, research has demonstrated that hematocrit levels are not uniform across the entire circulatory system [5].

Nevertheless, blood traverses the microcirculation, where vessels have diameters of only a few micrometers. In this context, the cells are comparable in size to the vessels, rendering the notion of blood viscosity less applicable. This is because blood cannot be likened to a uniform solution or suspension [12].

In such situations, the factors influencing flow resistance include plasma viscosity, deformability and concentration of red blood cells, and the forces responsible for maintaining the cohesion of red cell rouleaux. Additionally, the size of red blood cells is a factor that can be considered unrelated to rheology. It’s important to highlight that young cell discharged from the bone marrow into the bloodstream measure 120 fl, and as they age, they undergo changes in membrane, content, shape, and size concurrently [12].

1.1.2.WBC

Because of the small contribution of leukocytes in the total peripheral blood cell population, they are often neglected in the discussion of hemorheological concepts. Hence, under macroscopic, bulk flow conditions, the existence of leukocytes is generally overlooked. However, this does not diminish the significance of their mechanical properties or their potential impact on blood flow. Leukocytes constitute a notably diverse cell population, exhibiting variations in both quantity and quality. Despite maintaining the overall viscosity of blood, an elevated count of leukocytes can significantly impact microcirculation flow due to their larger and less flexible nature compared to red blood cells (RBCs) [23]. Moreover, during specific pathophysiological conditions like inflammation, there are rapid fluctuations in the quantity of leukocytes, and these cells undergo an activation process leading to significant structural and functional changes [24].

It is now apparent that these normal slow flowing leukocytes have the ability hold up and alter the flow of red blood cells through capillaries, impacting perfusion and resistance within the microcirculation [23][25][26].

Apart from the consequences of their mechanical stiffness, it’s noteworthy that during inflammation, leukocytes adhering to the post-capillary venule walls lead to elevated flow resistance, as they constrict the lumen without causing complete occlusion. The presence of altered leukocyte rheology in microvascular dysfunction was first identified in animal studies, particularly in the setting of myocardial ischemia induced by arterial ligation and hemorrhagic shock [27][28].

It’s intriguing to note that, despite their lower concentration compared to red blood cells (i.e., nearly a 3 orders of magnitude difference), microscopic studies have revealed that individual leukocytes exhibit a flow resistance in small vessels approximately three times greater than that of red blood cells [29].

The structure of leukocytes renders them considerably more resistant to deformation compared to red blood cells. Consequently, leukocytes exhibit a slow flow through capillaries, influencing the distribution of red blood cells. Additionally, the necessity for leukocytes to migrate into tissues allows them to actively alter their structure and, consequently, their mechanical properties. Reduced perfusion pressure, as observed in conditions like occlusive ischemia or shock, may result in the entrapment of resting primary leukocytes in capillaries. In such circumstances, cells may undergo activation in poorly perfused regions, undergoing changes in their mechanical and adhesive characteristics, and may not be flushed out after reperfusion or pressure recovery. Alternatively, systemic alterations in leukocyte rheology, induced by activated agents or cells released from inflamed or damaged tissue, or the presence of bloodborne pathogens (e.g., bacterial toxins or autoantibodies), may result in entrapment in susceptible tissues such as the lungs. Therefore, comprehending the structural foundation of leukocyte rheology and its regulation offers crucial insights into the physiological and pathological responses of these cells [30].

Leukocytes and platelets in circulation need to bind to the blood vessel walls to fulfill their respective immune defense and hemostatic functions. Inadequate recruitment or a lack of control in this process can lead to pathological consequences. It is now widely acknowledged that adhesion is influenced by the specific hemodynamic conditions at the local level and is regulated by the rheological characteristics of the blood. The speed of cell movement prior to capture and the shear forces exerted on them during adhesion play a crucial role in determining the effectiveness of attachment. The rheological properties of the blood directly impact these hemodynamic parameters [31].

1.1.3. PLT

From a hemorheological point of view, platelets are of minor significance, despite having internally complex contents with substantial viscosity. This is due to their much smaller size compared to red blood cells and leukocytes, with diameters ranging from 2-3 µm. In normal blood, their overall volume is even less than that of leukocytes. Consequently, platelets have minimal impact on both whole blood viscosity and microvascular resistance. Their principal function is to contribute to the hemostatic mechanism, playing a central role in the coagulation process during clotting [12].

1.2. Liquid phase

Plasma proteins hold hemorheological significance for two primary reasons. Firstly, due to their higher concentration, large size, and frequently asymmetrical shape, they exert a substantial influence on plasma viscosity. The second reason for their hemorheological importance is their ability to induce the loose aggregation of red blood cells, known as rouleaux. From a hemorheological perspective, rouleaux formation is crucial as it makes blood viscosity highly dependent on the shear rate to which it is exposed [12].

Plasma viscosity is chiefly influenced by the protein content, with different protein fractions making distinct contributions. Albumin, constituting approximately 60% of the total protein pool in plasma, contributes about 36% to the difference in viscosity between water and plasma [18]. Fibrinogen, despite representing only about 4% of the total protein weight in plasma, contributes around 22% to plasma viscosity under physiological conditions. Globulin fractions, with their higher molecular weight, contribute more significantly to plasma viscosity than albumin [5].

1.2.1. Proteins

Albumin constitutes approximately 36% of the variance in viscosity between water and plasma, yet it constitutes about 60% of the total protein pool in plasma [32]. Fibrinogen, a 340 kDa glycoprotein synthesized by liver cells, plays a crucial role in platelet aggregation, ultimately contributing to blood clot formation and the maintenance of hemostasis [33]. The variation in the relative contributions of these protein fractions arises from their molecular size and shape, with fibrinogen exhibiting significantly greater asymmetry compared to other proteins [32].

1.2.2. Ions

Approximately 1% of the plasma’s weight is composed of ions. Blood contains various ions, including Na+, Cl-, K+, Ca++, HCO3-, and PO4--. Among these, Na+ is the most concentrated cation, exerting significant osmotic influence. Precise regulation of Na+ concentration is crucial. Deviations from the normal range can cause red blood cells to either shrink or swell, impacting their mechanical properties and, consequently, blood viscosity. Another vital ion in terms of hemorheology is the anion HCO3-. Its significance lies in its role in regulating blood pH within the narrow normal range of 7.35 to 7.45. Any deviation from this range adversely affects the mechanical properties of red blood cells, thereby influencing their viscometric effects [12].

1.2.3. Metabolic molecules

Metabolic molecules, such as glucose, urea, and amino acids, typically possess molecular weights in the range of a few hundreds of Daltons. Together, they constitute approximately 1% of the plasma by weight. Unlike salts, the concentrations of these molecules are somewhat less rigorously controlled. For instance, the accepted normal ranges for glucose and urea are 0.7 to 1.0g/l and 80 to 250mg/l (urea nitrogen), respectively. These molecules generally exert minor hemorheological effects, and thus, further elaboration on them will not be provided here [12].

2. Evaluation of Blood Components Changes in MS Patients

2.1. Cellular phase

2.1.1.RBC

The hematocrit levels between the two groups of MS patients and controls showed no significant difference in the majority of studies [19][34][35][36]. However, Caimi et al. observed lower hematocrit levels in MS patients [35]. In a study where 27 MS patients were matched for sex and age (within 5 years) with individual control patients, except in one case where matching was not possible, similar hemoglobin levels were noted in both the MS group (mean 14.0 g/dl, range 11.6-16.4) and the controls (14.0 g/dl, 12.2-16.2). The mean corpuscular volume (MCV) was significantly higher in the MS group (mean 91.90 [SD 4.80] fl) compared to the controls (88.65 [4.13] fl) (P=0.011) and also exceeded the normal range for the laboratory (P<0.001) [37].

Furthermore, a percentage of MS patients (6.7%, 3.3%, and 10%, respectively) exhibited low levels of hemoglobin, hematocrit, and MCV [38].

Reported metabolic abnormalities of long-chain fatty acids in the red blood cell (RBC) membranes of MS patients have been documented [39][40]. RBC deformability, defined as the capacity of RBCs to reversibly deform under externally applied shear forces [41], is a crucial aspect of rheological properties [42]. Some studies indicate a significant difference in RBC deformability between MS patients and control groups [19][34], with observed abnormalities in MS erythrocyte morphology aligning with changes in blood rheology [19]. Increased whole blood viscosity in MS patients [34] is suggested as a factor contributing to impaired RBC deformability, although some studies report no difference in RBC deformability among MS patients [36].

Apart from RBC deformation during the activation of the inflammatory process in MS patients [14], impaired membrane fluidity of RBCs is also noted [21]. When both membrane fluidity and deformability are compromised, RBCs become susceptible to hydrolysis [43][44]. This condition is associated with a reduction in the number of RBCs and hematocrit in MS patients. Gloudina et al. demonstrated an inverse correlation between RBC count and the Kurtzke Expanded Disability Status Scale (EDSS), suggesting that a decrease in RBC counts was associated (though not significantly) with the worsening of symptoms in MS patients [35].

2.1.2. WBC

A series of studies conducted in human revealed compromised flow properties of circulating neutrophils in individuals with critical leg ischemia, intermittent claudication post-exercise-induced pain, and those in the recovery phase from myocardial infarction or stroke [45]. While these responses might be secondary to ischemic events, they have the potential to influence the progression or outcome of the respective diseases [30]. Alterations in granulocyte rheology can be triggered by autoantibodies known as anti-neutrophil cytoplasm antibodies (ANCA), associated with the pathology of small-vessel vasculitis. ANCA induce increased cell adhesiveness and rigidity, potentially impacting pathology in the lungs and kidneys. These responses are challenging to separate in vivo [46]. Numerous studies indicate elevated total white blood cell (WBC) counts and specific components of WBC, including neutrophils, basophils, and monocytes, in the peripheral blood of MS patients. Simpson et al. documented an increased leukocyte count in both male and female MS patients compared to controls [19].

At the time of diagnosis, individuals with MS exhibited higher counts of basophils and neutrophils compared to the healthy group, while the numbers of lymphocytes and eosinophils did not differ between the two groups [47]. Pierson et al. reported an apparent increase in the number of neutrophils in MS patients [48]. Similarly, Gloudina et al. demonstrated an elevated neutrophil percentage in MS patients, with a non-significant positive correlation with Cerebellar Functional Systems Scores (FSS) [35]. However, the immune cell composition in MS patients showed limited correlation with disease outcomes.

In the early phase of MS, Akaishi et al. discovered a strong association between the blood monocyte count (exclusive of other blood cells) and the clinical severity of MS [47].

2.1.3. PLT

Numerous studies have explored the platelet count and its correlation with the severity of MS symptoms. In one study, MS patients exhibited a significant increase in platelet numbers compared to controls (controls: median ± quartile range, 258 ± 88.0 × 10^9/l; patients: 292 ± 133 × 10^9/l; P=0.04) [35]. The reason for this elevation in platelets among patients remains unclear. Platelet activation can initiate a cascade of reactions leading to shape change, granule release, and aggregation, and increased platelet stickiness has been reported in MS patients.

Contrastingly, Sheremata and colleagues found no significant difference in platelet counts between the patient and control groups. However, their study revealed significant platelet activation in MS patients. Platelet-derived microparticles (PMP) were markedly elevated in MS (P<0.001), and CD62p expression showed a significant increase (P<0.001). Platelet-associated IgM, though not IgG, showed a marginal elevation in MS (P=0.01) [49]. The mechanisms behind this activation and its relevance to MS remain unknown [49]. In Morel’s investigation, despite the comparable average platelet levels in the control and patient groups, it was observed that isolated platelets from individuals with secondary progressive (SP) MS exhibited significantly stronger adhesion to typical subendothelial thrombogenic proteins—specifically collagen and fibrinogen—compared to platelets from healthy controls. Moreover, these patient-derived platelets displayed heightened reactivity to physiological agonists such as ADP (adenosine diphosphate) or collagen [50].

During an exacerbation of MS, there is an increased sensitivity of platelets to both ADP and noradrenaline [51]. Clinically, it has been established that platelets ultimately contribute to a more adverse disease outcome, as evidenced in measurements of MS patients [35] (Figure-2).

2.2. Liquid phase changes in MS

2.2.1. Proteins

Fibrinogen, recognized as an acute-phase reactant, exhibits an increased concentration during inflammatory processes such as MS [52]. Despite the elevation in whole blood viscosity observed in MS patients, Brunetti et al. surprisingly found that fibrinogen levels in MS patients were lower than normal [34].

Miranda Acuna et al. (2017) aimed to explore the relationship between plasma fibrinogen levels and the presence of active/visible lesions in MRI. They concluded that determining plasma fibrinogen levels during symptom recurrence holds a high positive predictive value.

Alternatively, the deposition of fibrinogen/fibrin in the central nervous system (CNS) has been widely employed as an indicator of blood-brain barrier (BBB) dysfunction in evaluating inflammatory diseases, including MS. Yates et al. (2017) discovered a significant over-representation of fibrin(ogen) deposition in the motor cortex of MS patients compared to healthy controls in all cellular compartments.

Sobel and Mitchell (1989) suggest that fibrinogen plays a role in the initial stages of inflammatory demyelination. Deposition in white matter precedes gadolinium leakage in active lesions, occurring prior to becoming visible on MRI [53].

Concerning fibrinogen levels in MS patients, varying findings have been reported. Ehling et al. (2011) concluded that fibrinogen levels were not elevated in both plasma and cerebrospinal fluid (CSF) compartments in MS patients. They observed a negative correlation between plasma viscosity and fibrinogen values (r=-0.38; t=2.43; P=<0.025) [34].

The aggregation of red blood cells (RBCs) is influenced by properties of both the suspending phase and cellular characteristics, which are known to be altered during physiopathological processes. RBC aggregation occurs when RBCs are suspended in solutions containing macromolecules with sufficient molecular weight and hydrated size at an appropriate concentration. Fibrinogen, a crucial macromolecule in plasma, has strong correlations with the extent of RBC aggregation.

The concentration of fibrinogen influences several factors associated with red blood cell aggregation, such as aggregate size, yield

stress, low-shear viscosity of RBC suspensions, and the rate of erythrocyte sedimentation [55][56][57][58][59].

Fibrinogen can also serve as a biomarker for disease activity. Zhang et al. (2016) demonstrated elevated fibrinogen levels in both neuromyelitis optica (NMO) and MS compared to the control group, with a positive correlation with the number of lesion segments (r=0.259). In NMO, a significant positive correlation was observed between fibrinogen levels and an increased Expanded Disability Status Scale (EDSS) (r=0.265).

Additional macromolecules like α2-macroglobulin, IgM, and IgG have been shown to influence red blood cell (RBC) aggregation in plasma. However, their impact on RBC aggregation becomes apparent only at concentrations significantly higher than physiological levels [54].

## Discussion

As we found out, blood rheology depends on its components. In the cellular part of blood, red blood cells play a more important role than platelets and white blood cells due to their higher concentration. [12]. In MS, lesions involve a primary rheological abnormality characterized by reduced flexibility of erythrocytes. According to the Poiseuille equation, disturbances in blood rheology increase the likelihood of stasis in the smallest veins, where shear rate is at its lowest. This diminished microcirculatory flow can lead to ischemic tissue damage.

An inflammatory response to tissue damage may contribute to the abnormal permeability of the blood-brain barrier in MS [55][56], along with the edema

frequently observed in early lesions. This edema, stemming from inadequate tissue blood supply, appears to be a complication linked to other MS symptoms, such as visual problems.

There is no study that directly examines the role of hemorheological variables in patients with MS. Also, there are many factors that affect hemorheology directly and indirectly, which cannot be reviewed, for example, ions and Metabolic Molecules affect hemorheology but their effect is very small and sometimes immeasurable.

On the other hand, the change of some variables in the blood flow causes an increase and the change of some leads to a decrease in blood viscosity, when these two changes happen together, the viscosity may not change much.

For instance, the bulk viscosity of blood may remain unchanged due to a reduction in red blood cell count combined with an increase in leukocytes.

Also, changes in blood flow and reduction in blood supply to tissues sometimes occur for reasons other than changes in viscosity. For instance, despite the bulk viscosity of blood remaining unchanged, an elevated count of leukocytes can significantly disrupt microcirculation flow due to the larger and more rigid nature of these cells compared to red blood cells. [23]Therefore, in this article, we have discussed the most important factors affecting hemorheology that can be measured, and the role of other factors is so small that it does not affect the results.

Also, we know that in addition to hemorheology, other factors such as the pressure created by the heart and possible vascular problems also affect the amount of blood perfusion in the tissues.

Conversely, alterations in certain blood flow variables can lead to an increase, while modifications in others can result in a decrease in blood viscosity. When these changes occur simultaneously, the overall viscosity may not undergo significant alterations. For instance, the bulk viscosity of blood may remain unchanged due to a reduction in red blood cell count combined with an increase in leukocytes.

Furthermore, changes in blood flow and reductions in blood supply to tissues can sometimes occur independently of viscosity changes. Even if the bulk viscosity of blood remains constant, an elevated number of leukocytes can profoundly impact microcirculation flow, given their larger and more rigid nature compared to red blood cells [23]. In this review, we analyzed various hemorheological factors in the context of MS, highlighting changes in blood viscosity, red blood cell count, and leukocyte count. Our findings indicate that while some variables lead to increases and others to decreases in blood viscosity, these changes can offset each other, resulting in minimal net alterations in overall viscosity. For example, a reduction in red blood cell count might be counterbalanced by an increase in leukocytes, leading to unchanged bulk viscosity.

Moreover, changes in blood flow and reductions in blood supply to tissues are sometimes independent of viscosity changes. Elevated leukocyte counts can significantly disrupt microcirculation due to their larger and more rigid nature compared to red blood cells, even if the bulk viscosity remains constant.

Despite these insights, there is a notable lack of direct studies examining hemorheological variables specifically in MS patients. While small factors like ions and metabolic molecules were considered, their effects are minimal and often immeasurable, which limits their inclusion in this review.

Beyond hemorheology, factors such as cardiac pressure and vascular issues also influence blood perfusion. Understanding how these factors interact with hemorheological variables can provide a more comprehensive view of blood flow dynamics in MS.

Future research should focus on direct examinations of hemorheological factors in MS and explore how these interact with other physiological factors to impact disease progression and symptom management.

## Conclusion

Finally, it can be concluded that according to the available evidence, the reduction of tissue blood perfusion can be considered as one of the causes of the development or exacerbation of MS disease. This reduction of blood supply can have various reasons.

For example, a decrease in the number of red blood cells that occurs during anemia or an increase in red blood cells that increases blood viscosity can both lead to a decrease in blood perfusion.

We need to conduct more detailed human studies to estimate hemorheological changes in MS, which will hopefully provide new solutions to the therapists of this disease.

## Conflict of Interest

The authors declare that they have no conflict of interest.
